# Effects of dynamic versus static parameter-guided fluid resuscitation in patients with sepsis: A randomized controlled trial

**DOI:** 10.12688/f1000research.147875.1

**Published:** 2024-05-22

**Authors:** Thiti Sricharoenchai, Pannarat Saisirivechakun

**Affiliations:** 1 Division of Pulmonary and Critical Care Medicine, Department of Medicine, Thammasat University, Pathum Thani, 12120, Thailand; 2 Department of Medicine, Nakhon Pathom Hospital, Nakhon Pathom, 73000, Thailand; 3 Department of Medicine, Faculty of Medicine, Nakhon Pathom Hospital, Nakhon Pathom, 73000, Thailand

**Keywords:** sepsis, dynamic parameter, static parameter, ultrasound, fluid resuscitation, mortality, norepinephrine, shock duration

## Abstract

**Background:**

Fluid resuscitation is an essential component for sepsis treatment. Although several studies demonstrated that dynamic variables were more accurate than static variables for prediction of fluid responsiveness, fluid resuscitation guidance by dynamic variables is not standard for treatment. The objectives were to determine the effects of dynamic inferior vena cava (IVC)-guided versus (vs.) static central venous pressure (CVP)-guided fluid resuscitation in septic patients on mortality; and others, i.e., resuscitation targets, shock duration, fluid and vasopressor amount, invasive respiratory support, length of stay and adverse events.

**Methods:**

A single-blind randomized controlled trial was conducted at Thammasat University Hospital between August 2016 and April 2020. Septic patients were stratified by acute physiologic and chronic health evaluation II (APACHE II) <25 or ≥25 and randomized by blocks of 2 and 4 to fluid resuscitation guidance by dynamic IVC or static CVP.

**Results:**

Of 124 patients enrolled, 62 were randomized to each group, and one of each was excluded from mortality analysis. Baseline characteristics were comparable. The 30-day mortality rates between dynamic IVC vs. static CVP groups were not different (34.4% vs. 45.9%, p=0.196). Relative risk for 30-day mortality of dynamic IVC group was 0.8 (95%CI=0.5-1.2, p=0.201). Different outcomes were median (interquartile range) of shock duration (0.8 (0.4-1.6) vs. 1.5 (1.1-3.1) days, p=0.001) and norepinephrine (NE) dose (6.8 (3.9–17.8) vs. 16.1 (7.6–53.6) milligrams, p=0.008 and 0.1 (0.1-0.3) vs. 0.3 (0.1-0.8) milligram⋅kilogram ^−1^, p=0.017). Others were not different.

**Conclusions:**

Dynamic IVC-guided fluid resuscitation does not affect mortality of septic patients. However, this may reduce shock duration and NE dose, compared with static CVP guidance.

## Trial registration

TCTR20160808002 in Thai Clinical Trials Registry ( https://www.thaiclinicaltrials.org/export/pdf/TCTR20160808002), Prospectively registered on 3 ^rd^ August 2016.

**List of abbreviations**
95%CI: 95% confidence interval
APACHE II: acute physiologic and chronic health evaluation II
BMI: body mass index
BW: body weight
cIVC: inferior vena cava collapsibility index
CI: confidence interval
cm: centimeters
CONSORT: Consolidated Standards of Reporting Trials
CVP: central venous pressure
dIVC: inferior vena cava distensibility index
Dmax: maximum diameter
Dmin: minimum diameter
e.g.: exempli gratia (for example)
et al.: et alia (and others)
etc.: et cetera (and so forth)
ETT: endotracheal intubation
F/U: follow-up
h: hour(s)
ICU: intensive care unit
i.e.: id est (that is)
IQR: interquartile range
IVC: inferior vena cava
kg: kilogram(s)
kg⋅m ^−2^
: kilogram(s) per square meter
KUB: kidney-ureter-bladder
L: liter(s)
LOS: length of stay
MAP: mean arterial pressure
min: minute(s)
mg: milligram(s)
mg⋅kg ^−1^
: milligram(s) per kilogram
mL: milliliter(s)
mL⋅kg ^−1^
: milliliter(s) per kilogram
mL⋅kg ^−1^⋅h ^−1^
: milliliter(s) per kilogram per hour
mmHg: millimeters of mercury
mmol⋅L ^−1^
: millimole(s) per liter
MV: mechanical ventilation
n: number
NaCl: sodium chloride
NE: norepinephrine
p: p-value
qSOFA: quick Sequential (Sepsis-Related) Organ Failure Assessment Score
SBP: systolic blood pressure
ScvO _2_
: central venous oxygen saturation
SD: standard deviation
SOFA: Sequential (Sepsis-Related) Organ Failure Assessment Score
SSC: Surviving Sepsis Campaign Guidelines
US: ultrasound
vs.: versus

## Introduction

Sepsis and septic shock potentially cause high mortality and burden for patients and health care system. ^1^^,^^2^ Rivers, et al. introduced early goal-directed therapy as the mainstay treatment of sepsis, which has been able to reduce the mortality rate of patients with sepsis or septic shock for more than two decades. ^3^ The Surviving Sepsis Campaign Bundle 2018 Update recommends to initiate rapid administration of intravenous crystalloid solution, at least 30 milliliters per kilogram (mL⋅kg ^−1^), in the first hour for resuscitation in the early phase of sepsis ^4^ because most patients with sepsis are affected by hypovolemic status. Adequate cardiac preload is an essential component for hemodynamic restoration in patients with sepsis. Nevertheless, excessive fluid resuscitation may occur and harm patients with sepsis. ^5^^–^^11^ Several physiologic studies have shown that dynamic variables, e.g., pulse pressure variation, ^12^^,^^13^ stroke volume variation, ^14^^–^^16^ respiratory change in aortic blood velocity, ^17^^,^^18^ vena cava diameter variation, ^19^^–^^22^ etc. were more accurate for assessment of fluid responsiveness, ^23^ while systematic reviews found that static central venous pressure (CVP) was not highly reliable for assessment of fluid responsiveness. ^24^^,^^25^ Surviving Sepsis Campaign Guidelines 2016 suggest using dynamic variables over static variables to guide fluid resuscitation, ^26^ but currently, dynamic variables are not yet standard for prediction of fluid responsiveness.

This study aimed to determine the effects of dynamic inferior vena cava (IVC)-guided versus static CVP-guided fluid resuscitation in patients with sepsis on mortality and other clinical outcomes, i.e., targets of resuscitation, duration of shock, amount of fluid and vasopressor administration, invasive respiratory support, length of stay (LOS) in hospital and intensive care unit (ICU), and adverse events.

## Methods

### Study design, setting and participant selection

This was a single-blind randomized controlled trial conducted at the emergency department, general medical wards and medical ICU of Thammasat University Hospital (TUH) between August 2016 and April 2020. The patients were screened if they met all of the following criteria: 1. age of 15 years or more; 2. clinical suspicion of infection with quick Sequential (Sepsis-Related) Organ Failure Assessment Score (qSOFA) of 2 or more ^27^; 3. evidence of organ dysfunction shown by Sequential (Sepsis-Related) Organ Failure Assessment Score (SOFA) of 2 or more ^27^; 4. unstable hemodynamics (systolic blood pressure (SBP) <90 millimeters of mercury (mmHg) or decrease in SBP >40 mmHg from baseline or mean arterial pressure (MAP) <70 mmHg) for 6 hours (h) or less; 5. fluid resuscitation required for unstable hemodynamics. The exclusion criteria were one or more of the following: 1. pregnant women; 2. cardiogenic pulmonary edema; 3. inability to lie down (e.g., scoliosis); 4. limited measurement of IVC diameter by ultrasound (US) (e.g., abdominal mass compressing IVC); 5. difficulty in performing central venous catheterization or CVP measurement (e.g., superior vena cava obstruction). The eligible patients who had given consent were included in the study. PS recruited and stratified patients by acute physiologic and chronic health evaluation II (APACHE II) of <25 or ≥25, then randomized them by blocks of 2 and 4 using sealed envelope ^TM^ program, to either measure IVC diameter variation by US or measure static CVP by central venous catheter and transducer in a ratio of 1:1, in order to guide fluid resuscitation. The allocation sequences in sealed envelopes were disclosed one by one when each patient was randomized. The process of fluid resuscitation guidance was covered from each patient by a screen.

### Intervention

***Static CVP measurement group***

The patients in the static CVP measurement group received central venous catheterization at right or left internal jugular vein by attending physicians. The first CVP measurement was done at the beginning of the study: CVP <8 mmHg and ≥8 mmHg were considered as fluid responsiveness and fluid non-responsiveness, respectively. Fluid-responsive patients were resuscitated by crystalloid solution (mostly 0.9% sodium chloride (NaCl) solution) 500 milliliters (mL) in 15 minutes (min), then CVP was measured repeatedly. Continuous fluid resuscitations and CVP measurements were performed alternately in the same manner until fluid non-responsiveness occurred (i.e., CVP ≥8 mmHg). Then patients with MAP <65 mmHg and fluid non-responsiveness received vasopressor(s) and/or inotropic agent(s) according to Surviving Sepsis Campaign Guidelines 2012, ^28^ intensive monitoring for 6 h, and were followed until shock recovery (i.e., MAP ≥65 mmHg and adequate tissue perfusion without vasopressor(s)), transfer or death within 30 days. Regardless of whether patients recovered from septic shock, they would receive a standard of care from TUH until discharge, transfer or death.

***Dynamic IVC measurement group***

**Figure 1.**
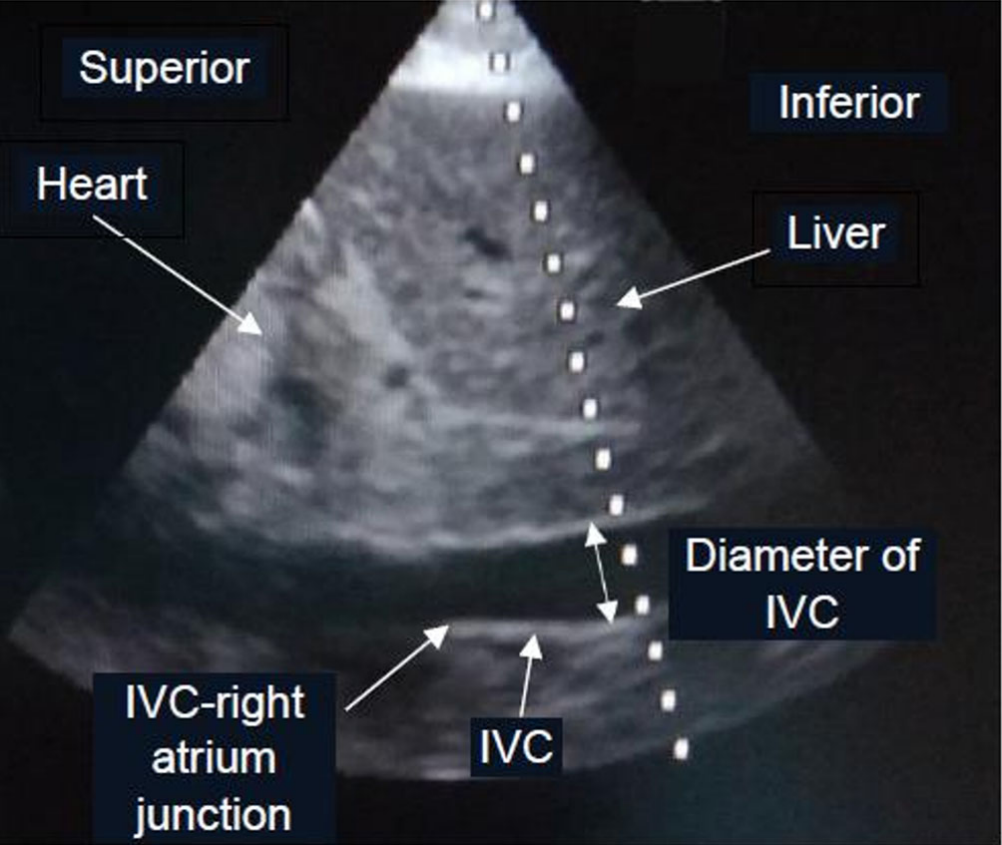
Ultrasonographic findings of IVC diameter measurement ^a^. Abbreviation: IVC, inferior vena cava. ^a^ The photo was taken by TS and was not used or previously published in any journal before.

**Figure 2.**
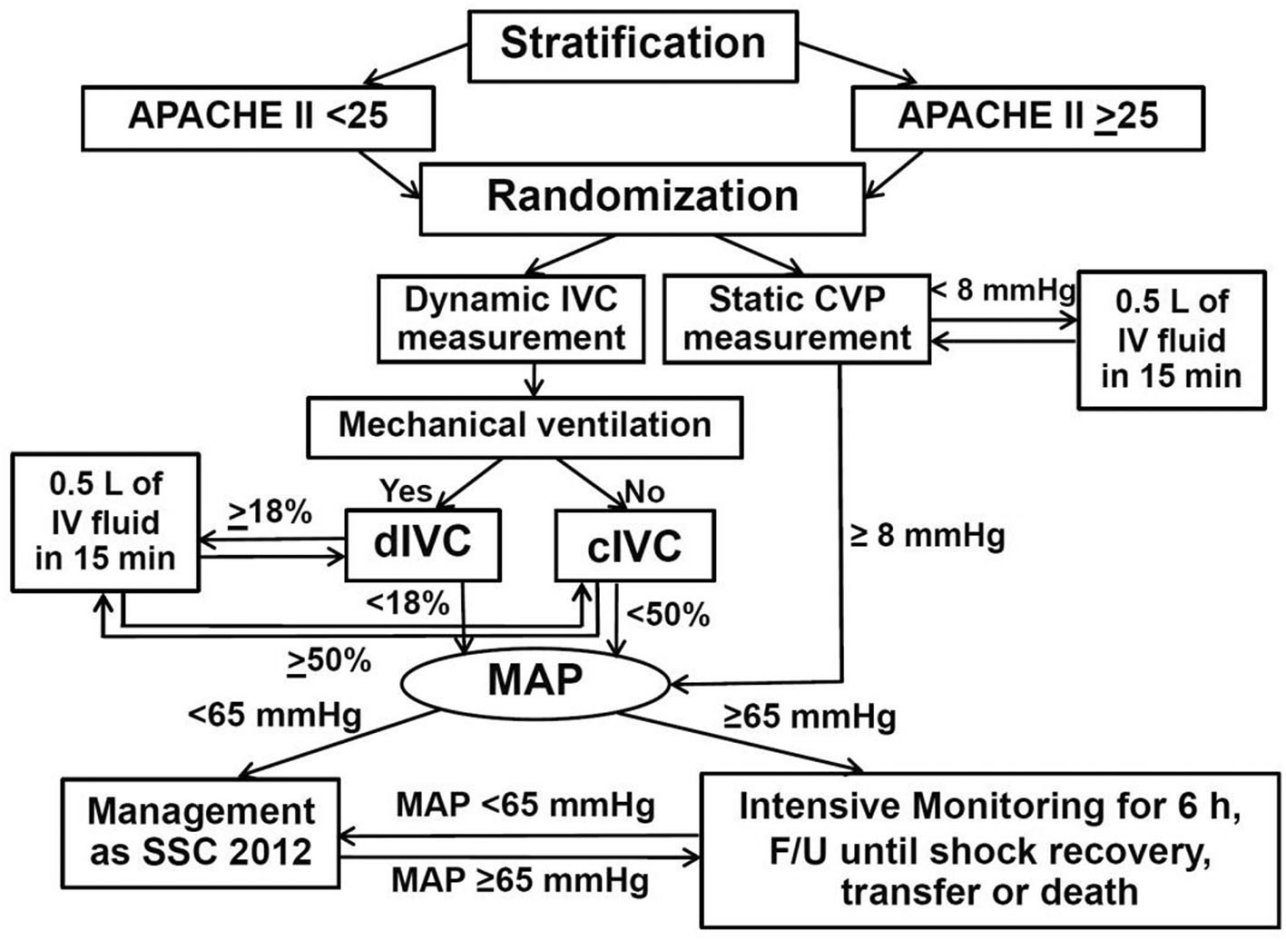
Flow diagram of study protocol. Abbreviations: APACHE II, acute physiologic and chronic health evaluation II; cIVC, inferior vena cava collapsibility index; CVP, central venous pressure; dIVC, inferior vena cava distensibility index; F/U, follow-up; h, hours; IV, intravenous; IVC, inferior vena cava; L, liter; MAP, mean arterial pressure; min, minutes; mmHg, millimeters of mercury; SSC, Surviving Sepsis Campaign Guidelines.

The patients in the dynamic IVC measurement group were divided into 2 subgroups: mechanically ventilated and spontaneously breathing. The US probe was placed subcostally in order to measure the IVC diameter at 3-4 centimeters (cm) from the IVC-right atrium junction ^29^ as Figure 1. while the patient was in a supine position. The first measurement of respiratory IVC diameter variation by US was done at the beginning of the study in both subgroups. Mechanically ventilated patients were measured for IVC distensibility index ((maximum diameter – minimum diameter)*100%/minimum diameter, (Dmax-Dmin)*100%/Dmin) with ≥18% and <18% considered as fluid responsiveness, and fluid non-responsiveness, respectively. ^30^ The maximum IVC diameter and minimum IVC diameter occurred during inspiration and expiration, respectively, in mechanically ventilated patients. Spontaneously breathing patients were measured for IVC collapsibility index ((Dmax-Dmin)*100%/Dmax) with ≥50% and <50% considered as fluid responsiveness, and fluid non-responsiveness, respectively. ^31^ In contrast, the maximum IVC diameter and minimum IVC diameter occurred during expiration and inspiration, respectively, for spontaneously breathing patients. Respiratory variation of IVC diameter measurement by US was used in this study because of feasibility and availability of US in our hospital settings. The measurement of respiratory variation of IVC diameter using US was performed by PS after training with 30 patients for 2 weeks and supervised by an intensivist. Dynamic IVC diameter measurement was done by using a LOGIQ C5 Premium Ultrasound Machine (GE Healthcare, Chicago, Illinois, USA). Sedative agents (midazolam and/or fentanyl) and neuromuscular blocking agents (atracurium or cisatracurium) were possibly administered in some mechanically ventilated patients to minimize respiratory effort, and maximize the validity of IVC distensibility index. Fluid-responsive patients were resuscitated continuously by crystalloid solution (also mostly 0.9% NaCl solution) 500 mL in 15 min, and IVC distensibility index or IVC collapsibility index measurements were done repeatedly and alternately until fluid non-responsiveness was achieved (i.e., IVC distensibility index <18% or IVC collapsibility index <50%) in the same way as the static CVP measurement group. The vasopressor(s) and inotropic agent(s) administration, including other treatment, monitoring and follow-up processes after the patients meeting fluid non-responsiveness until discharge, transfer or death within 30 days were similar to those of the static CVP measurement group. The flow diagram of study protocol is shown in Figure 2.

### Data collection and outcome measures

The patients’ data were recorded for baseline clinical characteristics (i.e., sex, age, weight, height, comorbidities), characteristics of sepsis (i.e., vital signs, confirmed or suspected source of infection, APACHE II at time of diagnosis), ICU admission, outcomes (i.e., survival status at 30 days after participation in the study, macrovascular targets (MAP, urine output) and microvascular targets (central venous oxygen saturation (ScvO _2_), serum lactate) at time of diagnosis of sepsis and 6 h later, time of treatment initiation and MAP ≥65 mmHg with adequate tissue perfusion, total volume of fluid administration in first 72 h of sepsis, accumulated dose of norepinephrine (NE), status of endotracheal intubation (ETT) with mechanical ventilation (MV) throughout study period, dates of initiation and termination of MV, dates of hospital admission and discharge, dates of ICU admission and discharge), and adverse events (i.e., fluid overload and pneumothorax in 30 days after participation in the study). The primary outcome was the 30-day mortality of patients; those who were discharged or transferred to other hospitals before 30 days were tracked for survival status at 30 days by telephone. The secondary outcomes were achievements of macrovascular targets (MAP ≥65 mmHg, urine output ≥0.5 milliliter(s) per kilogram per hour (mL⋅kg ^−1^⋅h ^−1^)) and microvascular targets (ScvO _2_ ≥70%, lactate clearance ≥10%) in the first 6 h, ^32^ duration of shock (time from treatment initiation to MAP ≥65 mmHg and adequate tissue perfusion), total volume of fluid administration and total volume of fluid administration per kilogram (kg) of body weight (BW) in the first 72 h, accumulated dose of NE and accumulated dose of NE per kg of BW, ETT with MV, MV duration, hospital LOS, ICU LOS and adverse events. The clinical outcomes were selected based on feasibility and standard practice of TUH.

### Statistical analysis

The sample size was estimated to be 122 patients (61 for each group) for the detection of 24% difference in 30-day mortality rate between the two intervention groups ^33^ with an alpha of 0.05 and a power of 80%. Interim analysis of efficacy was performed when the number of patients were recruited up to 50% of sample size. The trial would be terminated if 30-day mortality rates between two intervention groups were extremely different (Haybittle-Peto boundary with an alpha level of 0.002). Baseline clinical characteristics, primary and secondary outcomes were analyzed by descriptive statistics for all patients and each intervention group. Categorical variables were shown as proportion (percentage), and continuous variables were shown as mean ± standard deviation (SD) or median (interquartile range, IQR) as appropriate. Data of each intervention group were compared using Chi-square test for categorical variables, and using Independent two-sample t-test for normally distributed continuous variables or Mann-Whitney U test for non-normally distributed continuous variables. Univariable relative risk regression or univariable Poisson regression analysis was performed for binary outcomes to obtain the relative risk of dynamic IVC measurement-guided fluid resuscitation for each outcome, compared with static CVP measurement-guided fluid resuscitation. The 2-sided p-value (p) <0.05 was considered as statistical significance for comparison between the two groups and regression analysis. All data were analyzed using Stata SE, version 14.0 (StataCorp, College Station, Texas, USA), of which copyright license has been held by the Faculty of Medicine, Thammasat University. An open-access alternative software for statistical analysis is RStudio, which is available from https://posit.co/download/rstudio-desktop/.

### Ethical considerations

This study was conducted in accordance with The Code of Ethics of the World Medical Association (Declaration of Helsinki) for experiments involving human subjects. Ethics approval for research conduct, documentation of consent from each patient or their representative and information sheet was provided by the Human Research Ethics Committee of Thammasat University (Medicine) (Approval number: 127/2559) on 15 ^th^ July 2016. A written informed consent for the study was obtained from each patient or their legally authorized representative. No identifiable data of individual persons are presented. Consent for publication was a part of written informed consent for the study, which declared that no identifiable data of individual persons are presented.

## Results

**Figure 3.**
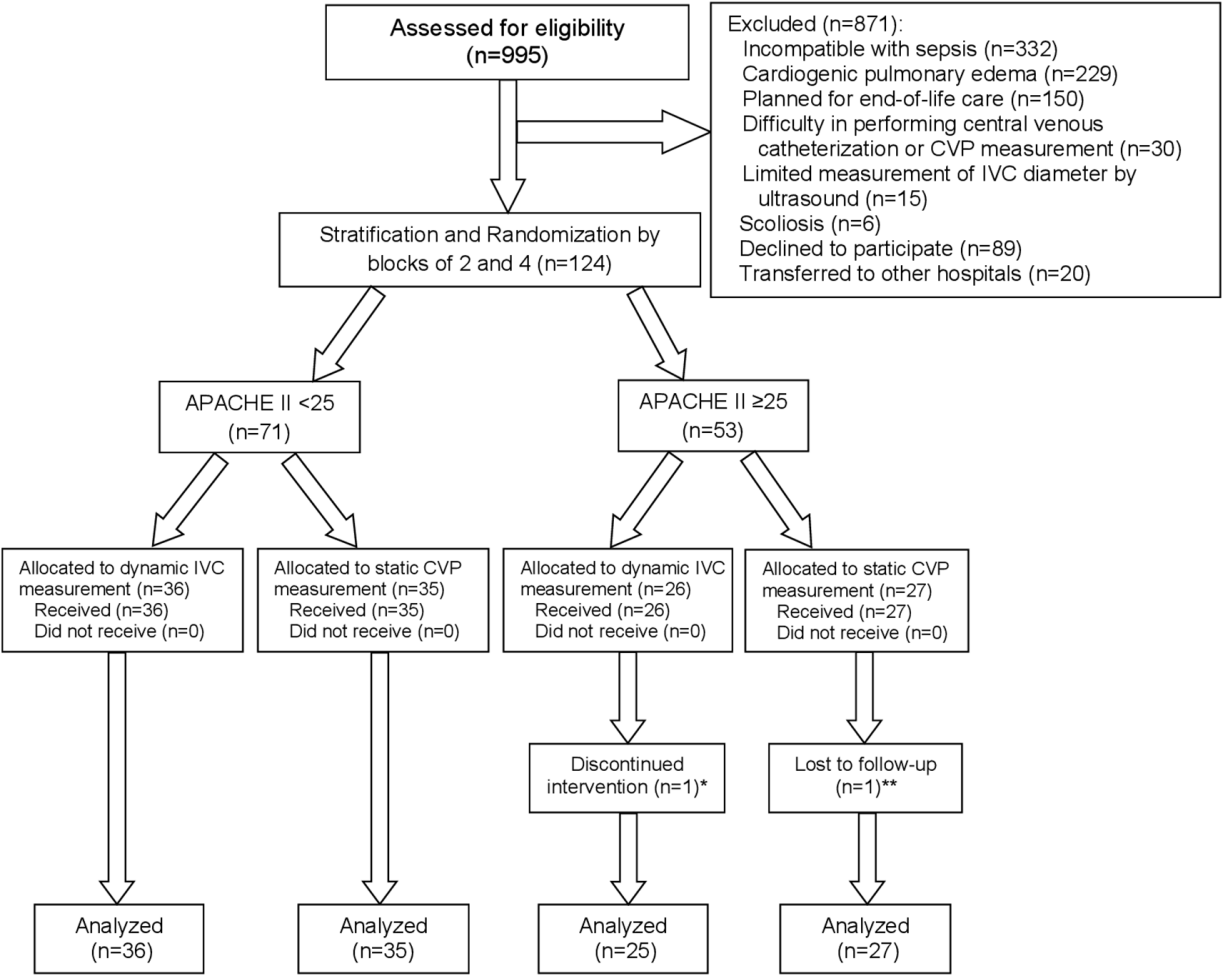
CONSORT flow diagram of study. Abbreviations: APACHE II, acute physiologic and chronic health evaluation II; CONSORT, Consolidated Standards of Reporting Trials; CVP, central venous pressure; IVC, inferior vena cava. *One patient withdrew informed consent because of discomfort for data collection. **One patient was transferred to other hospital on the same day of enrollment in the study, and their survival status at 30 days could not be tracked.

A total of 995 patients with sepsis or septic shock treated at the emergency department or medical wards in TUH between August 2016 and April 2020 were screened for eligibility, and 124 patients were included. After stratification by APACHE II of <25 or ≥25, 62 patients were randomized into each intervention group. Individual patients were followed until shock recovery, transfer or death within 30 days. One patient in the dynamic IVC measurement group withdrew after randomization because of discomfort for data collection, leaving 123 patients included in the modified intention-to-treat analysis. One additional patient in the static CVP measurement group was excluded from analysis for the 30-day mortality because they were referred to another hospital and their survival status could not be tracked. The study process from enrollment to analysis is shown as a Consolidated Standards of Reporting Trials (CONSORT) flow diagram in Figure 3. The study was ended after completion of patient recruitment and follow-up.

The baseline characteristics and severity of sepsis, including ICU admission, were not significantly different between the two intervention groups, as shown in Table 1. Of 111 patients (90.2%) who required NE, the number of patients in the dynamic IVC measurement group (52, 85.3%) was lower than that in the static CVP measurement group (59, 95.2%), but not statistically different (p = 0.064). The primary outcome and most secondary outcomes were not significantly different between both groups, i.e., mortality rates in 30 days, achievement of macrovascular target(s) (MAP ≥65 mmHg or urine output ≥0.5 mL⋅kg ^−1^⋅h ^−1^ or both) in 6 h or achievement of microvascular target(s) (ScvO _2_ ≥70% or lactate clearance ≥10% or both) in 6 h, as well as total volume of fluid and total volume of fluid per kg of BW administered in the first 72 h of sepsis, ETT with MV, duration of MV, hospital LOS and ICU LOS. However, the dynamic IVC measurement group demonstrated significantly shorter median (IQR) duration of shock, considerably lower median (IQR) accumulated dose of NE and lower median (IQR) accumulated dose of NE per kg of BW. Rates of adverse events were not different between the two intervention groups. All outcomes in detail are shown in Table 2. Thirty-day mortality rates and significantly different secondary outcomes (i.e., duration of shock, accumulated dose of NE and accumulated dose of NE per kg of BW) are presented in Extended data Figure S1., S2., S3. and S4. All findings were based on analyses of the dataset from this study. ^34^

**Table 1.** Baseline characteristics of patients.

Characteristics	All patients (n = 123)	Dynamic IVC measurement (n = 61)	Static CVP measurement (n = 62)
Age (years)	72.0 (61.0 – 80.0)	74.0 (64.0 – 81.0)	68.5 (55.0 – 78.0)
Male, n (%)	66 (53.7)	32 (52.5)	34 (54.8)
Height (cm)	160.8 (±8.0)	160.8 (±8.7)	160.9 (±7.4)
Weight (kg)	57 (50 – 60)	54 (50 – 60)	60 (50 – 61)
BMI (kg⋅m ^−2^)	22.0 (19.5 – 24.0)	21.4 (19.5 – 23.9)	22.3 (20.3 – 24.0)
qSOFA	2.4 (±0.5)	2.4 (±0.5)	2.3 (±0.5)
Initial SOFA	7.7 (±3.7)	6.9 (±3.2)	8.4 (±4.0)
APACHE II score	23 (20 – 29)	23 (17 – 28)	24 (20 – 30)
Initial MAP (mmHg)	60 (53 – 72)	60 (51 – 70)	60 (53 – 73)
Initial MAP <65 mmHg, n (%)	72 (58.5)	39 (63.9)	33 (53.2)
Initial ScvO _2_ (%) ^a^	70.1 (±10.9)	70.7 (±11.3)	69.8 (±10.7)
Initial lactate (mmol⋅L ^−1^)	3.3 (1.9 – 6.3)	3.0 (1.8 – 5.2)	4.1 (2.1 – 7.0)
**Comorbidities, n (%)**			
Diabetes mellitus	47 (38.2)	20 (32.8)	27 (43.6)
Hypertension	72 (58.5)	31 (50.8)	41 (66.1)
Coronary artery disease	15 (12.2)	7 (11.5)	8 (12.9)
Cerebrovascular disease	25 (20.3)	11 (18.0)	14 (22.6)
Chronic obstructive pulmonary disease	6 (4.9)	4 (6.6)	2 (3.2)
Chronic kidney disease	17 (13.8)	6 (9.8)	11 (17.7)
**Source of infection, n (%)**			
Respiratory system	47 (38.2)	19 (31.2)	28 (45.2)
Abdomen	28 (22.8)	19 (31.2)	9 (14.5)
KUB system	23 (18.7)	13 (21.3)	10 (16.1)
Skin & soft tissue	8 (6.5)	5 (8.2)	3 (4.8)
Neurological system	1 (0.8)	0 (0.0)	1 (1.6)
Systemic infection	4 (3.3)	1 (1.6)	3 (4.8)
Musculoskeletal system	1 (0.8)	1 (1.6)	0 (0.0)
Other sources	1 (0.8)	0 (0.0)	1 (1.6)
Unknown source	10 (8.1)	3 (4.9)	7 (11.3)
ICU admission, n (%)	34 (27.6)	17 (27.9)	17 (27.4)

Abbreviations: APACHE II, acute physiologic and chronic health evaluation II; BMI, body mass index; cm, centimeters; CVP, central venous pressure; ICU, intensive care unit; IVC, inferior vena cava; kg, kilograms; kg⋅m ^−2^, kilograms per square meter; KUB, kidney-ureter-bladder; MAP, mean arterial pressure; mmHg, millimeters of mercury; mmol⋅L ^−1^, millimole(s) per liter; n, number; qSOFA, quick Sequential (Sepsis-Related) Organ Failure Assessment Score; ScvO _2_, central venous oxygen saturation; SOFA, Sequential (Sepsis-Related) Organ Failure Assessment Score.

Initial ScvO _2_ and initial lactate were central venous oxygen saturation and serum lactate at time of diagnosis of sepsis, respectively.

Categorical data and continuous data were presented as number (%) and median (interquartile range, IQR) or mean (±standard deviation, SD) as appropriate, respectively.

^a^ This characteristic was analyzed from 101 patients (39 in Dynamic IVC measurement group, and 62 in Static CVP measurement group) who received central venous catheterization.

**Table 2.** Clinical outcomes of patients.

Outcomes	All patients (n = 123)	Dynamic IVC measurement (n = 61)	Static CVP measurement (n = 62)	p-value	Relative risk (95%CI)	p-value
**Primary outcome**						
**30-day mortality** ^**a**^ **, n (%)**	49 (40.2)	21 (34.4)	28 (45.9)	0.196 ^$^	0.8 (0.5 – 1.2)	0.201 ^α^
**Secondary outcomes**						
**Macrovascular target(s) achievement in 6 h**						
**MAP ≥65 mmHg, n (%)**	111 (90.2)	54 (88.5)	57 (91.9)	0.524 ^$^	1.0 (0.7 – 1.4)	0.842 ^β^
**MAP (mmHg)**	77.9 (±10.9)	78.6 (±11.7)	77.3 (±10.2)	0.528 ^@^		
**Urine ≥0.5 mL⋅kg** ^**-1**^ **⋅h** ^**-1**^ **, n (%)**	83 (67.5)	43 (70.5)	40 (64.5)	0.479 ^$^	1.1 (0.9 – 1.4)	0.480 ^α^
**Urine (mL⋅kg** ^**-1**^ **⋅h** ^**-1**^ **)**	0.8 (0.3 – 1.5)	0.8 (0.4 – 1.6)	0.8 (0.2 – 1.4)	0.345 ^#^		
**≥1 target, n (%)**	117 (95.1)	58 (95.1)	59 (95.2)	0.984 ^$^	1.0 (0.7 – 1.4)	0.996 ^β^
**2 targets, n (%)**	77 (62.6)	39 (63.9)	38 (61.3)	0.762 ^$^	1.0 (0.8 – 1.4)	0.762 ^α^
**Microvascular target(s) achievement in 6 h**						
**ScvO**_**2**_ **≥70%** ^**b**^ **, n (%)**	67 (66.3)	30 (76.9)	37 (59.7)	0.074 ^$^	1.3 (1.0 – 1.7)	0.063 ^α^
**ScvO _2_ (%)** ^**b**^	73.2 (±9.4)	75.2 (±8.1)	72.0 (±9.9)	0.090 ^@^		
**Lactate clearance ≥10%, n (%)**	73 (59.4)	36 (59.0)	37 (59.7)	0.941 ^$^	1.0 (0.7 – 1.3)	0.941 ^α^
**Lactate clearance (%)**	15.7 (0.0 – 41.3)	14.3 ((-11.1) – 41.0)	19.0 (0.0 – 41.3)	0.962 ^#^		
**≥1 target** ^**c**^ **, n (%)**	102 (89.5)	49 (94.2)	53 (85.5)	0.130 ^$^	1.1 (0.7 – 1.6)	0.623 ^β^
**2 targets** ^**d**^ **, n (%)**	38 (34.6)	17 (35.4)	21 (33.9)	0.866 ^$^	1.0 (0.6 – 1.8)	0.866 ^α^
**Other outcomes**						
**Total fluid volume in first 72 h (mL)** ^**e**^	8,599.5 (±2,899.2)	8,327.3 (±2,968.7)	8,929.4 (±2,822.5)	0.381 ^@^		
**Total fluid volume per kg of BW in first 72 h (mL⋅kg** ^**-1**^ **)** ^**e**^	154.8 (±59.9)	154.3 (±61.8)	155.5 (±58.4)	0.934 ^@^		
**Duration of shock (days)** ^**e**^	1.3 (0.7 – 2.4)	0.8 (0.4 – 1.6)	1.5 (1.1 – 3.1)	0.001 ^#^ *		
**Accumulated dose of NE (mg)** ^**f**^	10.8 (5.6 – 27.1)	6.8 (3.9 – 17.8)	16.1 (7.6 – 53.6)	0.008 ^#^ *		
**Accumulated dose of NE per kg of BW (mg⋅kg** ^**-1**^ **)** ^**f**^	0.2 (0.1 – 0.6)	0.1 (0.1 – 0.3)	0.3 (0.1 – 0.8)	0.017 ^#^ *		
**ETT with MV, n (%)**	75 (61.0)	33 (54.1)	42 (67.7)	0.121 ^$^	0.8 (0.6 – 1.1)	0.126 ^α^
**Duration of MV (days)** ^**g**^	7.5 (3.0 – 34.5)	5.0 (3.0 – 27.0)	16.0 (4.0 – 44.0)	0.190 ^#^		
**Hospital LOS (days)** ^**e**^	18.0 (9.0 – 44.0)	16.5 (6.5 – 36.5)	23.0 (11.0 – 51.0)	0.097 ^#^		
**ICU LOS (days)** ^**h**^	12.5 (6.0 – 33.0)	7.5 (5.5 – 30.0)	20.5 (8.0 – 49.0)	0.291 ^#^		
**Adverse events**						
**Fluid overload, n (%)**	17 (13.8)	6 (9.8)	11 (17.7)	0.204 ^$^	0.6 (0.2 – 1.4)	0.214 ^α^
**Pneumothorax, n (%)**	0 (0)	0 (0)	0 (0)	-	-	-

Abbreviations: BW, body weight; CI, confidence interval; CVP, central venous pressure; ETT, endotracheal intubation; h, hours; ICU, intensive care unit; IVC, inferior vena cava; LOS, length of stay; MAP, mean arterial pressure; mg, milligrams; mg⋅kg ^−1^, milligram(s) per kilogram; min, minutes; mL, milliliters; mL⋅kg ^−1^, milliliter(s) per kilogram; mL⋅kg ^−1^⋅h ^−1^, milliliter(s) per kilogram per hour; mmHg, millimeters of mercury; MV, mechanical ventilation; n, number; NE, norepinephrine; ScvO _2_, central venous oxygen saturation.

Lactate clearance was calculated by (serum lactate at time of diagnosis of sepsis – serum lactate at 6 h later) *100%/serum lactate at time of diagnosis of sepsis.

Categorical data and continuous data were presented as number (%) and median (interquartile range, IQR) or mean (±standard deviation, SD) as appropriate, respectively.

Data were analyzed using the following methods:

^$^ Chi-square test.

^@^ Independent two-sample t-test.

^#^ Mann-Whitney U test.

^α^ Relative risk regression.

^β^ Poisson regression.

* Statistically significant different.

^a^ This outcome was analyzed from 122 patients (61 in Dynamic IVC measurement group, and 61 in Static CVP measurement group) because one patient in static CVP group was transferred to other hospital and their survival status at 30 days could not be tracked.

^b^ This outcome was analyzed from 101 patients who received central venous catheterization (39 in Dynamic IVC measurement group, and 62 in Static CVP measurement group).

^c^ This outcome was analyzed from 114 patients who can be assessed for achievement of ≥1 microvascular target in 6 hours (52 in Dynamic IVC measurement group, and 62 in Static CVP measurement group).

^d^ This outcome was analyzed from 110 patients who can be assessed for achievement of 2 microvascular targets in 6 hours (48 in Dynamic IVC measurement group, and 62 in Static CVP measurement group).

^e^ These outcomes were analyzed from 73 survived patients (40 in Dynamic IVC measurement group, and 33 in Static CVP measurement group).

^f^ These outcomes were analyzed from 65 survived patients who required norepinephrine (33 in Dynamic IVC measurement group, and 32 in Static CVP measurement group).

^g^ This outcome was analyzed from 32 survived patients who required endotracheal intubation and mechanical ventilation (15 in Dynamic IVC measurement group, and 17 in Static CVP measurement group).

^h^ This outcome was analyzed from 22 survived patients who were admitted to ICU (12 in Dynamic IVC measurement group, and 10 in Static CVP measurement group).

## Discussion

Our study showed that the baseline characteristics were not different between the two intervention groups. The 30-day mortality rates were not different between both groups, nor were achievement of macrovascular and microvascular targets in 6 h, total volume of fluid administration and total volume of fluid administration per kg of BW in the first 72 h of sepsis, rate of ETT with MV, duration of MV, hospital LOS, ICU LOS and rates of adverse events. However, patients in the dynamic IVC measurement group experienced shorter duration of shock, lower accumulated dose of NE and lower accumulated dose of NE per kg of BW, compared with those in the static CVP measurement group.

To our knowledge, this is the first study attempting to determine the clinical effects of fluid resuscitation guided by feasible and practical hemodynamic parameter for prediction of fluid responsiveness in patients with sepsis or septic shock in Thailand. There was no difference in the 30-day mortality rates between the two groups, which was consistent with the 28-day mortality rate of septic shock patients in a study from Richard, et al. ^33^ Although the 30-day mortality rates in the two groups were not statistically different, the percentage of mortality in the dynamic parameter group was somewhat lower (absolute difference about 10%) than in the static parameter group in our study, and was even considerably lower (absolute difference about 20%) in that study. ^33^ This may imply important clinical significance in certain clinical situations, especially for the disease with high prevalence like sepsis, because the absolute number of deaths would be magnified by the prevalence of the disease. The mortality rates (at 30 days, ^35^^,^^36^ 90 days, ^37^ 180 days, ^35^ and hospital mortality ^38^) of surgical ICU patients between fluid therapy guided by dynamic variables and standard care (fluid therapy guided by static variables or clinical examination) were also not different in other studies. However, a recent systematic review and meta-analysis including studies of critically ill adult patients requiring acute fluid resuscitation demonstrated that dynamic variable-guided fluid therapy decreased the mortality risk (risk ratio = 0.59, 95%CI = 0.42-0.83, p = 0.002) compared with standard care. ^39^ The difference between our findings and the systematic review findings in terms of mortality may be partially explained by the different types of patients and therefore different pathophysiologies of diseases. The majority of patients in our study were medical patients while most patients in that systematic review were surgical patients. Medical patients usually have more comorbidities, which poses a higher risk of complications and/or death, compared with surgical patients, and this factor may interfere with the effect of intervention on mortality.

Shorter duration of shock and lower accumulated dose of NE (either total dose or total dose per kg of BW) in the dynamic IVC measurement group in our study suggests that dynamic IVC diameter variation may be able to predict fluid responsiveness better than static CVP value, so the patients received more adequate volume of fluid administration. This assumption corresponds to the study findings from Lanspa, et al., which showed that IVC collapsibility index threshold of ≥50% had a fair positive predictive value (75%) and a good negative predictive value (80%) for prediction of fluid responsiveness, although this did not reach statistical significance (p = 0.09). ^31^ Theoretically, patients with adequate fluid volume administration can likely maintain macrovascular and microvascular targets, and minimize duration of shock without excessive dose of vasopressors, resulting in fewer complications from prolonged shock or high dose of vasopressors. ^40^^–^^42^ However, patients in the dynamic IVC measurement group did not achieve more macrovascular or microvascular target(s) in 6 h than those in the other group in our study. The reason for this may be the fact that the timing of vasopressor initiation was not controlled because it was decided based on the individual attending physician’s discretion. Given that a higher proportion of patients who received vasopressor earlier could possibly restore macrovascular and microvascular targets as demonstrated in a study from Permpikul, et al. (CENSER trial), ^43^ this may confound the effect of fluid administration guidance method on achievement of macrovascular and microvascular targets.

In our study, total fluid volume and total fluid volume per kg of BW administered in the first 72 h were not different between the two intervention groups, which was consistent with a study from Trof, et al. ^44^ but different from a study by Richard, et al. which showed lower daily intravenous fluid volume administration in a dynamic variable-guided fluid therapy group. ^33^ The reason for different findings can be partially explained by different time-frames of fluid administration and also different dynamic parameters guiding fluid resuscitation between our study and that study. ^33^ Moreover, our study protocol did not control fluid administration after shock recovery occurring within 72 h, which affected total fluid volume and total fluid volume per kg of BW in the first 72 h. Another study by Douglas, et al. demonstrated less positive fluid balance at 72 h or ICU discharge in septic patients who received dynamic stroke volume-guided fluid resuscitation, compared to those with standard usual care, ^45^ but this outcome cannot be directly compared to total fluid volume in 72 h of our study.

Our study found no difference in rates of ETT and MV between the two intervention groups. This was a reasonable finding because indications of intubation and MV are broader than only unstable hemodynamics. Durations of MV were also not different between the two intervention groups in our study, which was the same as studies of Richard, et al., ^33^ Douglas, et al. ^45^ and Trof, et al., specifically for septic shock patients. ^44^

Hospital LOS in both intervention groups were not different, which was similar to some studies ^35^^,^^45^^–^^47^ but different from others (shorter hospital LOS in dynamic variable-guided fluid therapy group). ^36^^,^^48^^–^^52^ ICU LOS were not different between our two intervention groups, which also was consistent with several studies ^33^^,^^37^^,^^45^^,^^46^^,^^49^ but different from others. ^47^^,^^51^^,^^52^ The heterogeneous findings in hospital LOS and ICU LOS were likely caused by the different nature of diseases, given that most patients in these studies were various types of surgical patients, and there were specific indications for ICU admission for certain types of surgical patients (e.g., postoperative observation).

There are some limitations in this study. Firstly, the sample size was quite small and possibly underpowered to be able to detect the differences of some secondary outcomes between the two intervention groups. A study with a larger sample size is warranted to clarify whether such outcomes are different. However, our sample size was comparable or even greater than other studies assessing the effects on clinical outcomes between dynamic and static hemodynamic parameters. ^33^^,^^44^^,^^53^ Second, treatment of sepsis for each patient was based on individual attending physicians, except for fluid resuscitation and intensive monitoring in the early phase of sepsis. This might have interfered with the effects of intervention, but clinical practices were standardized by following the Surviving Sepsis Campaign Guidelines ^4^^,^^26^^,^^28^ and standard of care at TUH under supervision of specialists. Third, the US measurement process of respiratory variation of IVC diameter is operator-dependent. Accordingly, all US measurements of IVC diameter variation were done by the same well-trained physician and the same US machine to eradicate interobserver variability and standardize the findings of US measurement. Fourth, the parameters for prediction of fluid responsiveness in our study were derived from right-sided cardiac preload, which indirectly reflects the variation of stroke volume or cardiac output of the left ventricle after fluid administration. Nevertheless, right-sided cardiac preload-derived parameters were selected based on availability and practicability. Fifth, this was a single-center study, which may not be able to perfectly generalize the findings to other institutes or other hospitals. Physicians need to carefully consider the context of individual situations before adoption of our study findings for clinical practices. Strengths of this study include study design (randomized controlled trial), which could balance baseline characteristics and severity of sepsis in patients between the two intervention groups in order to minimize confounding factors affecting the outcomes, and feasibility for limited-resource hospitals.

## Conclusions

Dynamic IVC measurement-guided fluid resuscitation in patients with sepsis or septic shock does not affect mortality, macrovascular and microvascular targets, volume of fluid administration, ETT with MV, duration of MV, hospital LOS, ICU LOS and adverse events. Nevertheless, such intervention may decrease the duration of shock and accumulated dose of NE, compared with static CVP measurement-guided fluid resuscitation.

## Author contributions

Conceptualization: Sricharoenchai T. and Saisirivechakun P.; Data curation: Sricharoenchai T. and Saisirivechakun P.; Formal analysis: Sricharoenchai T. and Saisirivechakun P.; Investigation: Sricharoenchai T. and Saisirivechakun P.; Methodology: Sricharoenchai T. and Saisirivechakun P.; Project administration: Sricharoenchai T. and Saisirivechakun P.; Resources: Sricharoenchai T. and Saisirivechakun P.; Software: Sricharoenchai T. and Saisirivechakun P.; Supervision: Sricharoenchai T.; Validation: Sricharoenchai T.; Visualization: Sricharoenchai T. and Saisirivechakun P.; Writing − Original Draft Preparation: Sricharoenchai T.; Writing − Review & Editing: Sricharoenchai T. and Saisirivechakun P.

## Data Availability

Zenodo: Data for effects of dynamic vs. static parameter-guided fluid resuscitation in patients with sepsis, https://zenodo.org/records/10579408 or DOI: 10.5281/zenodo.10579407. ^34^ This project contains the following underlying data:
•
Data for dynamic vs. static parameter-guided resuscitation in sepsis.dta (baseline characteristics and outcomes of patients with sepsis) Data are available under the terms of the Creative Commons Attribution 4.0 International license (CC-BY 4.0). Zenodo: Supplementary materials for effects of dynamic vs. static parameter-guided fluid resuscitation in patients with sepsis, https://zenodo.org/records/10594154 (Sricharoenchai 2024) or DOI: 10.5281/zenodo.10594153. This project contains the following extended data:
•
Figure S1. Bar graphs showing the 30-day mortality rate. (30-day mortality rate between dynamic IVC measurement and static CVP measurement groups) •
Figure S2. Box and whisker plots showing the median (IQR) duration of shock. (median (IQR) duration of shock between dynamic IVC measurement and static CVP measurement groups) •
Figure S3. Box and whisker plots showing the median (IQR) accumulated dose of NE. (median (IQR) accumulated dose of NE between dynamic IVC measurement and static CVP measurement groups) •
Figure S4. Box and whisker plots showing the median (IQR) accumulated dose of NE per kg of body weight. (median (IQR) accumulated dose of NE per kg of body weight between dynamic IVC measurement and static CVP measurement groups) Data are available under the terms of the Creative Commons Attribution 4.0 International license (CC-BY 4.0). Zenodo: A completed CONSORT 2010 checklist for Effects of dynamic versus static parameter-guided fluid resuscitation in patients with sepsis: A randomized controlled trial, https://zenodo.org/records/10597483 (Sricharoenchai 2024) or DOI: 10.5281/zenodo.10597239. Data are available under the terms of the Creative Commons Attribution 4.0 International license (CC-BY 4.0).
