# Supplementary material for: Effects of dynamic versus static parameter-guided fluid resuscitation in patients with sepsis: A randomized controlled trial
Source: F1000Res. 2024 Jul 25;13:528. Originally published 2024 May 22. [Version 2] doi: 10.12688/f1000research.147875.2 (PMC11342037; doi:10.12688/f1000research.147875.2)
Supplement: Peer review history

# Reviewer report: Reviewer response for version 2

**DOI:** 10.5256/f1000research.169516.r306941

**Published:** 2024-08-22

**Authors:** Jhuma Sankar

**Affiliations:** 1 Division of Pediatric Pulmonology and Intensive Care, Department of Pediatrics, All India Institute of Medical Sciences, New Delhi, India

No further comments.

Is the work clearly and accurately presented and does it cite the current literature?

Yes

If applicable, is the statistical analysis and its interpretation appropriate?

I cannot comment. A qualified statistician is required.

Are all the source data underlying the results available to ensure full reproducibility?

Yes

Is the study design appropriate and is the work technically sound?

Yes

Are the conclusions drawn adequately supported by the results?

Yes

Are sufficient details of methods and analysis provided to allow replication by others?

Yes

Reviewer Expertise:

Septic shock; sepsis; hemodynamic monitoring; scoring systems; anti microbial resistance

I confirm that I have read this submission and believe that I have an appropriate level of expertise to confirm that it is of an acceptable scientific standard.

# Reviewer report: Reviewer response for version 2

**DOI:** 10.5256/f1000research.169516.r306940

**Published:** 2024-08-01

**Authors:** Surat Tongyoo

**Affiliations:** 1 Faculty of Medicine, Siriraj Hospital, Mahidol University, Bangkok, Thailand

Approved

Is the work clearly and accurately presented and does it cite the current literature?

Yes

If applicable, is the statistical analysis and its interpretation appropriate?

Yes

Are all the source data underlying the results available to ensure full reproducibility?

Yes

Is the study design appropriate and is the work technically sound?

Yes

Are the conclusions drawn adequately supported by the results?

Yes

Are sufficient details of methods and analysis provided to allow replication by others?

Yes

Reviewer Expertise:

Septic shock

I confirm that I have read this submission and believe that I have an appropriate level of expertise to confirm that it is of an acceptable scientific standard.

# Response

**Published:** 2024-08-01

**Authors:** Thiti Sricharoenchai

**Affiliations:** Department of Medicine, Thammasat University, Pathum Thani, Thailand

We are very grateful for your invaluable time, opinions and responses.

# Reviewer report: Reviewer response for version 1

**DOI:** 10.5256/f1000research.162119.r282206

**Published:** 2024-06-20

**Authors:** Surat Tongyoo

**Affiliations:** 1 Faculty of Medicine, Siriraj Hospital, Mahidol University, Bangkok, Thailand

I would like to support this article for approval in F1000Research, but after a minor revision. The following are my comments.

 1.Please explain why the author used CVP at 8 mmHg as a cut point for fluid responsiveness. For me it seem to be too low level for that. 

 2.Conventionally, we used follow up CVP with an elevation of CVP from baseline > 5-7 mmHg as a cut point for fluid responsive, why the author did not use this.

 3.For IVC US, author provide 2 cut point of IVC variation for fluid responsive, why you not provide different cut point for CVP as the positive intra-thoracic pressure might increase baseline CVP as well.

 4. Please provide more detail in your results manuscript section.

 5. Norepinephrine should be microgram/kg/min, not mg.

 6. Please discuss more about even patient in both group received fluid in same volume, why they received NE in different dose, and also the different in shock duration.

Is the work clearly and accurately presented and does it cite the current literature?

Yes

If applicable, is the statistical analysis and its interpretation appropriate?

Yes

Are all the source data underlying the results available to ensure full reproducibility?

Yes

Is the study design appropriate and is the work technically sound?

Yes

Are the conclusions drawn adequately supported by the results?

Yes

Are sufficient details of methods and analysis provided to allow replication by others?

Yes

Reviewer Expertise:

Septic shock

I confirm that I have read this submission and believe that I have an appropriate level of expertise to confirm that it is of an acceptable scientific standard, however I have significant reservations, as outlined above.

# Response

**Published:** 2024-07-22

**Authors:** Thiti Sricharoenchai

**Affiliations:** Department of Medicine, Thammasat University, Pathum Thani, Thailand

**Reviewer's Comment 1.** Please explain why the author used CVP at 8 mmHg as a cut point for fluid responsiveness. For me it seem to be too low level for that. 

**Authors' Response:** Thank you for your comment. The Surviving sepsis campaign guidelines 2012 (reference 28 of the manuscript) ^1^ recommended to keep CVP at least 8 mmHg to be adequate volume status. The CVP of 8 mmHg was used as the cut-off point for fluid responsiveness in other studies (reference 33 and 51 of the manuscript). ^2, 3^ This CVP cut-off point (8 mmHg) is also usually used in our hospital settings, most attending physicians are familiar with this CVP value for assessment of fluid responsiveness. This would help the process of study to be consistent for all patients in the static CVP group. The explanations were presented in Intervention of Methods section.

**Reviewer's Comment 2.** Conventionally, we used follow up CVP with an elevation of CVP from baseline > 5-7 mmHg as a cut point for fluid responsive, why the author did not use this.

**Authors' Response:** We appreciate your comment. While we agree that an elevation of CVP from baseline > 5-7 mmHg is usually considered as a cut-off point for fluid responsiveness, the elevation of CVP from baseline usually refers to the increased value of CVP after the patient receives fluid administration, which is more likely to be a dynamic parameter, not a static parameter as we intended to determine the effect of fluid resuscitation guidance. Therefore, we used the CVP of 8 mmHg as a cut-off point for assessment of fluid responsiveness.

**Reviewer's Comment 3.** For IVC US, author provide 2 cut point of IVC variation for fluid responsive, why you not provide different cut point for CVP as the positive intra-thoracic pressure might increase baseline CVP as well.

**Authors' Response**: We are grateful for your comment and concern. The Surviving sepsis campaign guidelines 2012 (reference 28 of the manuscript) ^1^ recommended to keep CVP of 8-12 mmHg regardless of respiratory variation of intrathoracic pressure. Our study followed this by considering the CVP of ≥ 8 mmHg as adequate volume status in the static CVP group, as well as the study by Richard, et al. (reference 33 of the manuscript). ^2^ The reasons were provided in Intervention of Methods section.

**Reviewer's Comment 4.** Please provide more detail in your results manuscript section.

 Authors' Response: We acknowledge your advice. ***We have revised your concerned issues in Methods, Results and Discussion sections.***

**Reviewer's Comment 5.** Norepinephrine should be microgram/kg/min, not mg.

**Authors' Response:** Thank you for your suggestion. We used the rate of norepinephrine as “microgram/kg/min”. However, we calculated further for the dose of norepinephrine to be “mg” as follows:

 1) Accumulated dose of norepinephrine received by each patient throughout the total vasopressor duration; the rate of norepinephrine (microgram/kg/min) multiplied by the body weight (kg) and the duration of norepinephrine administration (min), then divided by 1,000. Thus, the accumulated dose of norepinephrine was converted to “mg”.

 2) Accumulated dose of norepinephrine per kg of body weight received by each patient throughout the total vasopressor duration; the rate of norepinephrine (microgram/kg/min) was multiplied by the duration of norepinephrine administration (min), then divided by 1,000. Thus, the accumulated dose of norepinephrine was converted to “mg per kg”.

 The details of unit conversion of norepinephrine were shown in Data collection and outcome measures of Methods section.

**Reviewer's Comment 6**. Please discuss more about even patient in both group received fluid in same volume, why they received NE in different dose, and also the different in shock duration.

**Authors' Response**: We are very grateful for your comment. The total fluid volume administered in the first 72 hours in most patients included the volume of fluid during the shock period and after the resolution of shock until 72 hours, because durations of shock were less than 72 hours in majorities of both groups. Furthermore, fluid volume after the resolution of shock until 72 hours for individual patients depended on the attending physicians’ discretion and was not controlled in this study. Therefore, total fluid volume in the first 72 hours cannot reflect the efficacy of guidance methods for fluid resuscitation, and may not be related to the accumulated dose of norepinephrine and duration of shock. This was explained in Results and Discussion sections.

**References**

 1. Dellinger RP, Levy MM, Rhodes A, et al.: Surviving Sepsis Campaign: international guidelines for management of severe sepsis and septic shock, 2012. *Intensive Care Med.* 2013;39:165–228.

 2. Richard JC, Bayle F, Bourdin G, *et al.*: Preload dependence indices to titrate volume expansion during septic shock: a randomized controlled trial. *Crit. Care.* 2015; **19**:5.

 3. Goepfert MS, Richter HP, Zu Eulenburg C, et al.: Individually optimized hemodynamic therapy reduces complications and length of stay in the intensive care unit: a prospective, randomized controlled trial. *Anesthesiology.* 2013;119:824–836.

# Reviewer report: Reviewer response for version 1

**DOI:** 10.5256/f1000research.162119.r282204

**Published:** 2024-06-14

**Authors:** Jhuma Sankar

**Affiliations:** 1 Division of Pediatric Pulmonology and Intensive Care, Department of Pediatrics, All India Institute of Medical Sciences, New Delhi, India

Abstract: No comments 

 Introduction: Optimal 

 Methods: The effect size for calculation of sample size is too large 24%.  How was this number chosen? An ultrasound method to guide resuscitation may not be able to produce such a large difference in mortality. Ten % at the most would have been optimal.  This should be addressed as a limitation. 

 Results: No comments 

 Discussion: Please address the limitations.

Is the work clearly and accurately presented and does it cite the current literature?

Yes

If applicable, is the statistical analysis and its interpretation appropriate?

I cannot comment. A qualified statistician is required.

Are all the source data underlying the results available to ensure full reproducibility?

Yes

Is the study design appropriate and is the work technically sound?

Yes

Are the conclusions drawn adequately supported by the results?

Yes

Are sufficient details of methods and analysis provided to allow replication by others?

Yes

Reviewer Expertise:

Septic shock; sepsis; hemodynamic monitoring; scoring systems; anti microbial resistance

I confirm that I have read this submission and believe that I have an appropriate level of expertise to confirm that it is of an acceptable scientific standard, however I have significant reservations, as outlined above.

# Response

**Published:** 2024-07-22

**Authors:** Thiti Sricharoenchai

**Affiliations:** Department of Medicine, Thammasat University, Pathum Thani, Thailand

**Reviewer's Comment:** The effect size for calculation of sample size is too large 24%.  How was this number chosen? An ultrasound method to guide resuscitation may not be able to produce such a large difference in mortality. Ten % at the most would have been optimal.  This should be addressed as a limitation. 

**Authors' Response:** Thank you so much for your comment. The difference in mortality rate was selected based on the study findings from Richard, et al. (28-day mortality rates in preload dependence group vs. control group were 23% vs. 47%, respectively) (reference 33 of the manuscript). ^1^ However, we agreed that the difference in mortality rate of 24% was quite high. This issue was explained in the limitations of Discussion section.

 References

 1. Richard JC, Bayle F, Bourdin G, *et al.*: Preload dependence indices to titrate volume expansion during septic shock: a randomized controlled trial. *Crit. Care.* 2015; 19: 5.
